# Inhibition of *Pseudomonas aeruginosa* with a recombinant RNA-based viral vector expressing human β-defensin 4

**DOI:** 10.1186/s12866-014-0237-z

**Published:** 2014-09-27

**Authors:** Sehee Park, Jin Il Kim, Ilseob Lee, Joon-Yong Bae, Min-Woong Hwang, Donghwan Kim, Seok-Il Jang, Hyejin Kim, Mee Sook Park, Hyung-Joo Kwon, Jin-Won Song, Yong Suk Cho, Wook Chun, Man-Seong Park

**Affiliations:** Department of Microbiology, College of Medicine, and the Institute for Viral Diseases, Korea University, Seoul, 136-705 Republic of Korea; Department of Microbiology, College of Medicine, Hallym University, Chuncheon, Gangwon-do 200-702 Republic of Korea; Department of Burn Surgery, Burn Center, Hangang Sacred Heart Hospital, Hallym University Medical Center, Seoul, 150-719 Republic of Korea

**Keywords:** Burn wounds, Human β-defensin, Newcastle disease virus, *Pseudomonas aeruginosa*

## Abstract

**Background:**

Harassed with extensive epithelial burn wounds, patients can be affected by complications, such as infection, hypovolemic shock, hypothermia, and respiratory failure. Immediate first aid and followed supportive cares are critical for the prevention of severe complications. However, secondary bacterial infection is hard to be controlled in burn patients, and *Pseudomonas aeruginosa* (*P. aeruginosa*) is one of the top listed pathogens perturbing burn wounds beyond the antibiotics spectrum.

**Results:**

To find the way for efficacious protection from the pseudomonas-mediated complications in burn patients, we assessed the *in vitro* and *in vivo* inhibitory values of human β-defensin 4 (hBD4), which is known as a member of the cationic, antimicrobial peptides found in human cells of many kinds. The Newcastle disease virus (NDV) was used as a viral vector for the expression of hBD4 in burn wounds. Expressed from the recombinant NDV (rNDV-hBD4), hBD4 effectively inhibited the pseudomonal growths in cell culture media. In a mouse model, severely burn-injured skin was recovered by the direct installation of the rNDV-hBD4 infected cells in the burn wounds whereas that of control mice remained severely damaged.

**Conclusions:**

We suggest that the application of hBD4 may protect burn patients from secondary pseudomonal infection and provide a therapeutic potential for burn wound treatment.

## Background

An estimated 450,000 burn injuries were treated in hospitals and clinics in the United States in 2011 [1]. Approximately 10% of burn injury cases require hospitalization for intensive medical treatment, and bacterial infection is one of the major causes of burn-related deaths [2]. Because thermal injury of the normal skin barrier results in depression of local and systemic immune responses and results in host protein leakage into necrotic tissues, pathogenic microbes efficiently proliferate and colonize in burn wound areas [3]. Among the various species of bacteria isolated from burn wounds, *P. aeruginosa* represents one of the principal pathogens [4]. Gram-negative, naturally antibiotic-resistant *P. aeruginosa* persistently infects burn wounds, delays wound recovery, and subsequently interferes with clinical therapies [5]. Thus, it is important to both control *P. aeruginosa* complications in the early stages of burn injury and reduce the risk of septic shock in burn patients [6].

Management of burn wounds requires comprehensive measures. Fluid resuscitation, pain control, pulmonary care, nutritional support, surgical debridement, and other intensive treatments are important considerations for severe burn injuries [7,8]. Topical or systemic applications of antimicrobial agents have also been shown to protect patients with burn wounds from subsequent bacterial infections. However, the therapeutic effects of antibiotics against bacterial invasion appear to be limited, especially in severe cases of burn injuries [9], and antibiotic use often induces complications involving the development of antibiotic-resistant strains [10]. To be controlled, these resistant strains need even more and stronger measures of antibiotics, or damages may develop further into septic consequences. Controversy over antibiotics in burn patient treatments can be also seen in the fact that burn-injured children treated with antibiotics exhibit higher infection rates and poorer prognosis during sepsis than non-treated children [11]. Moreover, in cases of patients with a burn injury of more than 40% of the total body surface area, subsequent secondary bacterial infections are difficult to be controlled with antibiotic intervention alone [12].

Found in vertebrates and invertebrates, defensins which are small, cysteine-rich antimicrobial peptides may be an alternative measure against antibiotics-resistant bacteria [13]. Two main classes of defensins, α- and β-defensins, have been studied in the context of bacterial infections [14]. Among the many subfamilies of β-defensins, we focused on human β-defensin 4 (hBD4), which can be found in testes, epithelial surfaces of thyroid glands, lungs, uterus, and kidneys, because it displayed a strong antimicrobial activity, especially against *P. aeruginosa* [15-17]. However, due to the relatively small size of the hBD4 molecule, a stable, high-yield delivery platform is required for the successful expression of the protein in target tissues [18]. Recently, an adenovirus vector has been used to deliver human β-defensin 3 into normal human skin cells [19]. However, use of the adenoviral vector has been criticized for poor gene delivery efficacy and safety issues [20,21]. Anti-adenoviral patient antibodies might represent another hurdle preventing the clinical use of adenoviral vectors [22]. Instead, we utilized a *Paramyxoviridae* avian Newcastle disease virus (NDV). The NDV has been recognized for its efficacy as a RNA viral vector not only for cancer therapies [23] but also for vaccine constructs [24]. Approved safety and superior expression efficiency for the inserted foreign gene add another merit to the usefulness of the NDV vector in humans [25,26].

In this study, we present the feasibility of hBD4 for the protection of burn wounds from secondary pseudomonal manifestation. Inserted into the recombinant NDV construct, hBD4 was effectively delivered right into the burn-injured epithelial layer of mice, and promoted skin healing as complete as an unburned control even in the event of concurrent *P. aeruginosa* infection.

## Methods

### Ethics statements

All animal procedures were conducted in accordance with the recommendations in the Guide for the Care and Use of Laboratory Animals of the Animal, Plant, and Fisheries Quarantine and Inspection Agency of Korea, and the experimental protocol was approved by the Institutional Animal Care and Use Committee of Hallym University (permit number: Hallym 2009-16).

### Cells and viruses

Human lung epithelial A549, human laryngeal cancer HEp-2, Madin-Darby canine kidney (MDCK), and Madin-Darby bovine kidney (MDBK) cells were obtained from the American Type Culture Collection (ATCC; Manassas, VA). Primary human dermal fibroblast (HDF) cells were obtained from Modern Cell and Tissue Technologies (Seoul, Republic of Korea). Primary chicken embryo fibroblast (CEF) cells were established from fertile chicken eggs (9-10 days old). Cells were maintained in appropriate media and supplemented with 10% fetal bovine serum (FBS; Hyclone, Logan, UT), 100 U/ml penicillin, and 100 μg/ml streptomycin (Invitrogen, Carlsbad, CA).

Newcastle disease viruses were propagated in fertilized chicken eggs, and the viral presence was confirmed by hemagglutination assay (HA assay) using 0.5% (v/v) chicken erythrocytes and by sequence analysis using reverse transcriptase-PCR (RT-PCR) method.

### Generation of recombinant NDV expressing hBD4 (rNDV-hBD4)

Human beta-defensin 4 (hBD4, NCBI accession: AJ314834) was amplified by PCR using testes cDNAs of the Human Total RNA Master Panel II (Clontech, Mountain view, CA). The sequences of primers are 5′-GCAGCCCCAGCATTATGCAG-3′ for sense and 5′-AAGCTACTGAGG TCCTACTT-3′ for anti-sense. After sequence confirmation, hBD4 was inserted into a specific restriction enzyme site, *Sac*II, between *P* and *M* genes of the NDV/Lasota (NCBI accession: JF950510) to generate the NDV-hBD4 plasmid (pNDV-hBD4). A set of NDV primers, 3013P (5′-GGCCGCGGTTAGAAAAAATACGGGTAGAACACTAGTCCGCCACCATGCAGAGACTTGTGCTG-3′) and 3430M (5′-GGCCGCGGAAATCAGGGTTTTGTACGATT-3′ for the anti-sense primer) were used to confirm pNDV-hBD4 construction. To rescue the recombinant NDV-hBD4 (rNDV-hBD4) virus, pNDV-hBD4 and supporting plasmids (pTM/Lasota-NP, -P, and -L) were transfected into A549 cells. 24 h later, transfected cells were co-cultured with CEF cells and incubated for 72 h at 37°C, 5% CO_2_. Co-cultured cell supernatants were inoculated into fertilized chicken eggs for rNDV-hBD4 propagation.

### GFP expression levels in various cells by recombinant NDV infection

A549, HEp-2, MDCK, MDBK, HDF, and CEF cells were seeded into 12-well plates at a density of 1.0 × 10^5^ cells/well. With the approximately 80 ~ 90% confluent cell density, cells were infected with the recombinant NDV expressing green fluorescent protein (rNDV-GFP) at the same multiplicity of infection (MOI = 1) for 1 h. At 20 h post-infection (hpi), expressed GFP intensity was measured in triplicate.

### Immunofluorescence confocal assay

To detect hBD4 expression, MDBK cells (1.0 × 10^5^ cells per well) were cultured directly on the 24-well plates with glass coverslips and infected with the rNDV-hBD4 at an MOI of 1 for 1 h. At 12 hpi, Golgi stop protein transport inhibitor (BD Bioscience, San Jose, CA) was treated for 10 h. The cells were fixed with 4% paraformaldehyde and permeabilized with 0.2% Triton X-100 in PBS (PBST). After treated with PBST containing bovine serum albumin (PBST-BSA), NDV proteins and expressed hBD4 were detected primarily with polyclonal rabbit anti-NDV antibody and monoclonal mouse anti-hBD4 antibody, respectively. Then, Texas Red conjugated anti-rabbit antibody (Jackson ImmunoResearch, West Grove, PA) and Alexa 488 conjugated anti-mouse antibody (Invitrogen) were used as secondary antibodies. Hoechst 33258 (Invitrogen) was used for cell nucleus detection. The cells were imaged with a LSM 510 Meta LNO laser scanning microscope (Carl Zeiss, Jena, Germany) at Korea Basic Science Institute, Chuncheon, Korea. PBS-infected samples were used as a control.

### Quantification of hBD4 with ELISA

To quantify hBD4 expression, MDBK cells were infected with the rNDV-hBD4 at an MOI of 10. At 24 hpi, cell supernatants were collected and prepared with the Centrifugal Filter (Millipore, Billerica, MA) for 1:10, 1:50, and 1:100 dilutions. hBD4 was then quantified in triplicate using polyclonal rabbit anti-hBD4 antibody and hBD4 peptide (Ana Spec, San Jose, CA) by enzyme-linked immunosorbent assay (ELISA). Cell culture media and rNDV-infected cell supernatants were used as controls.

### *In vitro* inhibition efficacy of hBD4 against *P. aeruginosa*

MDBK cells were infected with the rNDV-hBD4 at an MOI of 10 for 1 h, and cell supernatants were collected at 12 and 24 hpi, respectively. The cell supernatants were then mixed with the same volume of *P. aeruginosa* (1 × 10^7^ colony forming unit, cfu), which was obtained from Korean Culture Center for Microorganisms (Seoul, Republic of Korea; KCCM #11803; originated from MDB strain BU 277, ATCC#10145), and the mixtures were incubated at 37°C, 5% CO_2_ for 6 h. Each mixture was spread onto LB agar plate and incubated at 37°C, 5% CO_2_ for 16 h. *P. aeruginosa* colonies were counted using ChemiDoc XRS + (Bio-Rad, Hercules, CA). Cell culture media and rNDV-infected cell supernatants were used as controls.

### *In vivo* inhibition efficacy of hBD4 against *P. aeruginosa* in burn-injured mouse skin

To make burn wounds, BALB/c mice (female, 6-week-old; NARA Biotech, Seoul, Republic of Korea) were anesthetized with the combination of xylazine (Narcoxyl, InterVet, The Netherlands) and zoletil (Zoletil 50, Virvac, France). Mice were then shaved on their back skin and burned with a square-shaped brass pole (16 mm × 16 mm), being heated up to 100°C and sterilized by an alcohol lamp for ~10 seconds. Right after burn procedure, the damaged skin of mice was infected with 50 μl of *P. aeruginosa* (1.0 × 10^4^ cfu) via subcutaneous injection. For the expression of hBD4 in the burn wounds, MDBK cells were infected with the rNDV-hBD4 at an MOI of 10. At 12 hpi, the cells (~5 × 10^6^ cells/mouse) were injected subcutaneously in the burn-injured, *P. aeruginosa*-infected skin of mice. The rNDV-infected MDBK cells were used for the treatment of a control mouse group. Mice were grouped as B (affected by burn), B/P (affected by burn and *P. aeruginosa*), B/P/N (affected by burn, *P. aeruginosa*, and rNDV-infected MDBK cell), and B/P/H (affected by burn, *P. aeruginosa*, and rNDV-hBD4-infected MDBK cell).

### Statistics

Statistical significance of GFP expression in cells (A549 vs. MDBK) and hBD4 expression (mock vs. rNDV-hBD4) was assessed by unpaired, two-tailed Student’s *t* test (^*^, *p* < 0.05; ^**^, *p* < 0.01; and ^***^, *p* < 0.001). One-way ANOVA was applied to that of the colony count of *P. aeruginosa* (mock vs. rNDV-hBD4 or B/P vs. B/P/H) and skin recovery (B/P vs. B/P/H) (^*^, *p* < 0.05; ^**^, *p* < 0.01; and ^***^, *p* < 0.001) results and confirmed by Tukey’s multiple comparison test.

## Results

### Generation of the rNDV-hBD4 virus

**Figure 1:**
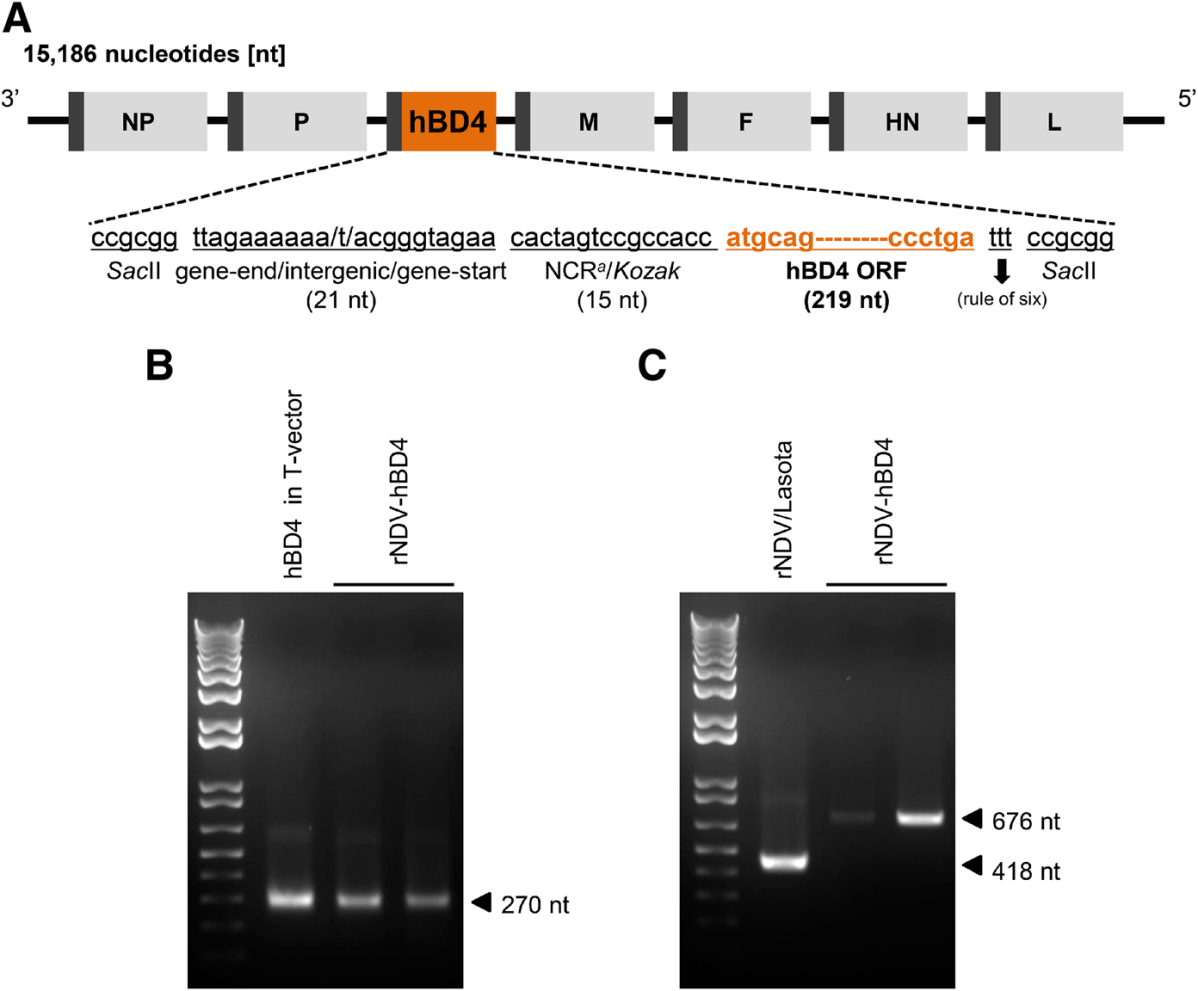
**Generation of the rNDV-hBD4 virus. (A)** Construction of the full-length NDV-hBD4 plasmid (pNDV-hBD4). **(B)** After rescuing the virus via reverse genetics, the forward and reverse hBD4 primers were used for hBD4 detection (the PCR product was ~270 nucleotides in length). **(C)** The 3013P forward and 3430M reverse primers were used for detecting the region between the C-terminal P gene and the N-terminal M gene of the rNDV-hDB4. An hBD4 T-vector and wild-type pNDV/Lasota were used as controls.

We first engineered a full-length plasmid of the NDV/Lasota strain (pNDV-Lasota) as a viral vector to express hBD4 (pNDV-hBD4) (Figure 1A). Using the constructed pNDV-hBD4, we rescued the rNDV-hBD4 virus by plasmid-based reverse genetics as described previously [27]. At 3 days post-transfection, HA positive samples were inoculated into fertilized chicken eggs for the propagation of the rNDV-hBD4 virus. To confirm the rescue of the rNDV-hDB4 virus, the egg allantoic fluids were used for the reverse transcriptase-PCR (RT-PCR). The hBD4-inserted T vector was used as a control. When using the hBD4 primer set (see Methods, Generation of recombinant NDV expressing hBD4 section), we detected the hBD4 nucleotide (nt) size of PCR products in two stocks of the rNDV-hBD4 virus and in the hBD4-inserted T vector control (Figure 1B). However, the NDV 3013P and 3430M primers set yielded ~ 670 nt size RT-PCR products only in the rNDV-hBD4 samples due to the insertion of hBD4 between NDV P and M genes whereas only ~400 nt size product was detected in the wild-type (wt) NDV/Lasota virus (Figure 1C). These results confirmed the right insertion of hBD4 into the NDV virus and the generation of the rNDV-hBD4 virus.

### hBD4 expression from the rNDV-hBD4 virus

**Figure 2:**
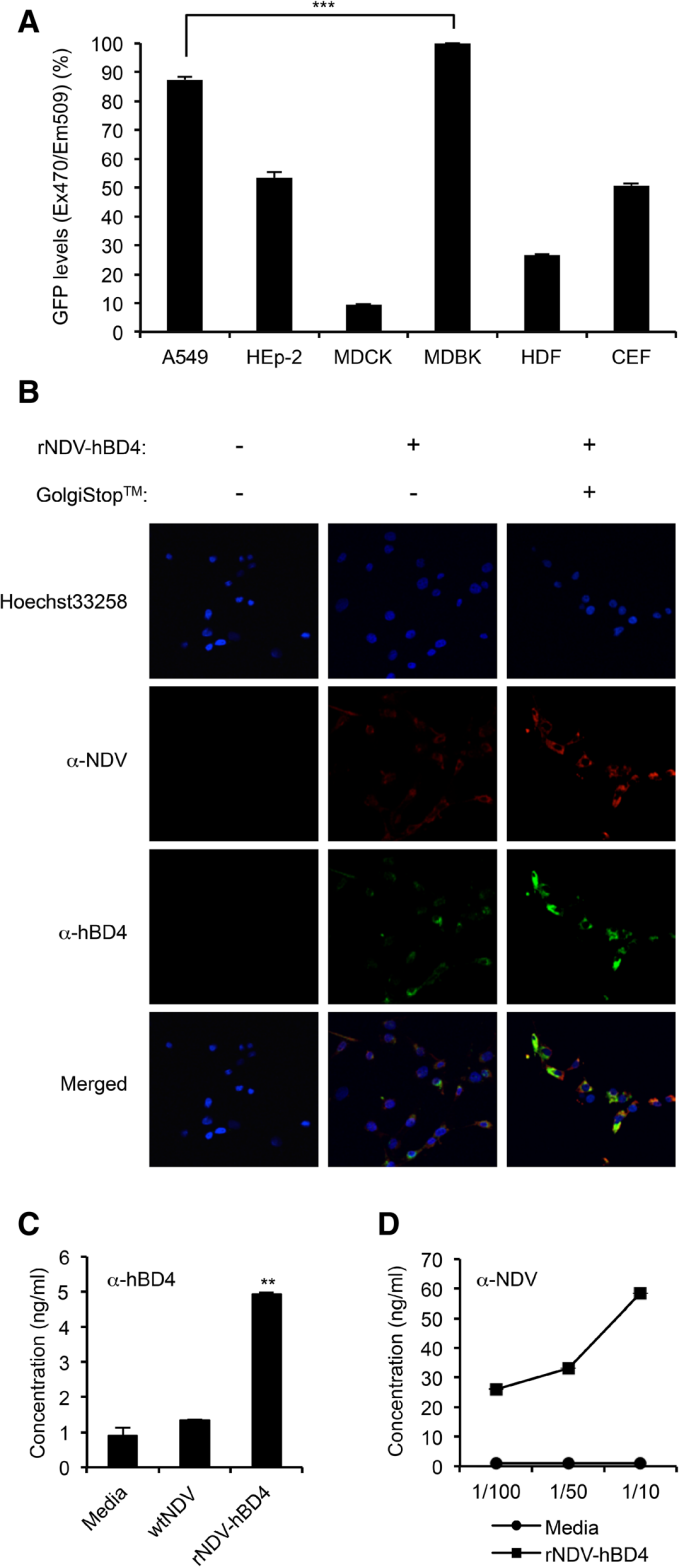
**Evaluation of hBD4 expression in rNDV-hBD4-infected cells. (A)** A549, HEp-2, MDCK, MDBK, HDF, and CEF cells were infected with the rNDV-GFP virus and assessed for GFP expression (^***^, *p* < 0.001). **(B)** MDBK cells were infected with the rNDV-hBD4 and treated with GolgiStop™, and hBD4 (green), NDV proteins (red), and the cell nuclei (blue) were detected in a confocal microscopy. **(C)** MDBK cells were infected with the rNDV-hBD4, and the expressed hBD4 in the cell supernatants was quantified at 24 hpi by ELISA (^**^, *p* < 0.01). **(D)** The expressed NDV proteins were also quantified and the mock-infected cell supernatants were used as a control.

Burn-damaged skin may not support sustained hBD4 expression after the rNDV-hBD4 infection. Hence, we needed an intermediate host suitable for NDV infection and hBD4 expression. Using the rNDV-GFP virus, we screened various cells. Among 6 different cells tested (including CEF and HDF primary cells), MDBK cells expressed the strongest GFP signals (Figure 2A). In the subsequent experiment only using MDBK and A549 cells, better GFP signals were determined in MDBK cells than in A549 cells (data not shown). Based on these results, we decided to use MDBK cells for the rNDV-hBD4 application against the pseudomonal attack in burn injury.

We then detected hBD4, expressed in MDBK cells after rNDV-hBD4 infection. To trap expressed hBD4 inside the cells, MDBK cells were treated with GolgiStop™ protein transport inhibitor. As seen in a PBS-infected control, hBD4 was barely detectable without GolgiStop treatment (Figure 2B). However, GolgiStop treatment possibly inhibited hBD4 secretion into cell supernatants, and hBD4 was well detected along in the rNDV-hBD4-infected MDBK cells (Figure 2B). These results indicated hBD4 could be expressed from rNDV-hDB4 virus infection in MDBK cells.

However, hBD4 was also detected in the mock-infected (~1.5 ng/ml) and rNDV-infected cell supernatants (Figure 2C). As mentioned above, defensins can be found in various kinds of cells of living organisms. This might explain why hBD4 was detected in controls. To verify whether hBD4 expression was increased by rNDV-hBD4 virus infection, we determined the amount of NDV proteins using the same cell supernatants of Figure 2C. In 10-, 50-, and 100-fold diluted cell supernatants, we detected the proportional increases of NDV proteins whereas no trace of NDV was detected in the mock-infected control (Figure 2D). Considered together, these results indicate that hBD4 can be well expressed into the cell supernatants from rNDV-hBD4 virus infection in MDBK cells.

### Inhibition efficacy of hBD4 against *P. aeruginosa*

**Figure 3:**
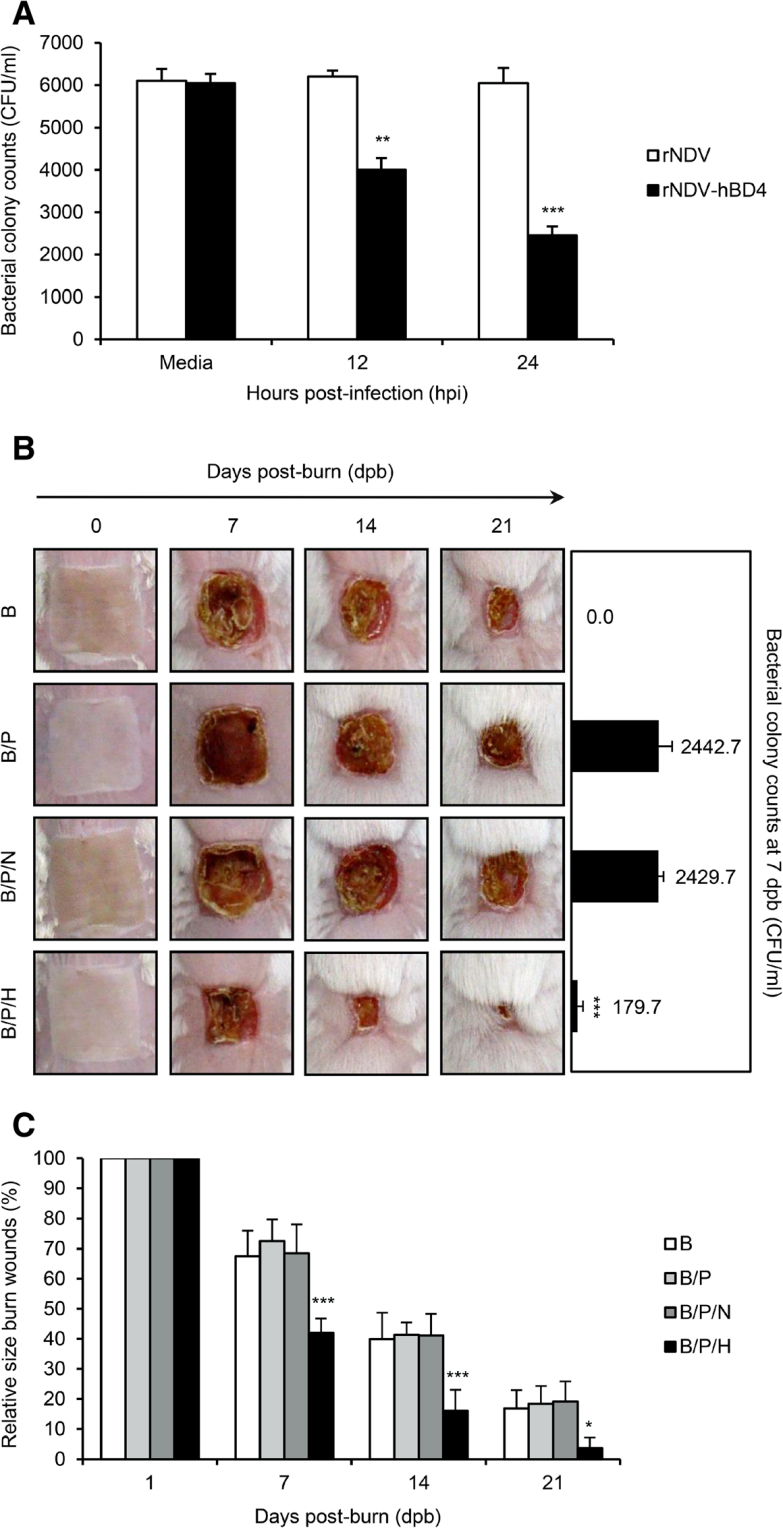
**Inhibitory efficacy of the rNDV-hBD4 virus against** ***P. aeruginosa*** **infection in burn wounds in a mouse model. (A)** After infection with the rNDV-hBD4, MDBK cell supernatants were collected at 12 or 24 hpi and mixed with *P. aeruginosa* (1.0 × 10^4^ cfu) at 37°C. The number of *P. aeruginosa* colonies in penicillin-free LB agar plates was quantified and compared with the number of colonies in the mock-infected samples (^**^, *p* < 0.01; ^***^, *p* < 0.001). **(B)** The back skin of mice was burned using a heated square brass pole (B group). *P. aeruginosa* infection was added in the B/P mice group, and the B/P/H mice group was treated with the rNDV-hBD4-infected MDBK cells. Mice treated with rNDV-infected MDBK cells were used as a control group. Representative photos from each group were presented for comparison. At 7 days post-burn, skin swab samples were plated onto cetrimide agar plates to determine *P. aeruginosa* colony counts (^***^, *p* < 0.001). **(C)** Skin recovery was determined by averaging the relative burn wound area and compared with those of the B group (100%) (^*^, *p* < 0.05; ^***^, *p* < 0.001).

Next, the *in vitro* inhibitory efficacy of hBD4 was evaluated against *P. aeruginosa*. To inhibit the growth of *P. aeruginosa* in cell culture media, we added the rNDV-hBD4-infected MDBK cell supernatants (MOI = 10, collected at 12 and 24 hpi) directly into the penicillin-free RPMI 1640 media, in which 10^7^ cfu of *P. aeruginosa* would be growing. We then counted the number of colonies after spreading *P. aeruginosa*-hBD4 mixture onto penicillin-free LB agar plates. Surprisingly, 33.88% and 59.50% reduction rates in the number of *P. aeruginosa* colonies were determined with the respective 12 (4.00 × 10^3^ cfu/ml) and 24 (2.45 × 10^3^ cfu/ml) hpi supernatants of rNDV-hBD4-infected MDBK cells, compared with that (6.05 × 10^3^ cfu/ml) of the mock-infected cell supernatant (Figure 3A). This meant that more amounts of hBD4 might be expressed into the cell supernatant at 24 hpi than at 12 hpi and that hBD4 effectively inhibited the growth of *P. aeruginosa*.

To assess the *in vivo* inhibition efficacy of hBD4 against *P. aeruginosa* in the burn wounds, we used a surrogate burn injury mouse model. Mice were grouped as B (burned, n = 6), B/P (burned and *P. aeruginosa*-infected, n = 6), B/P/N (burned, *P. aeruginosa*-infected, and rNDV-MDBK injected, n = 6), and B/P/H (burned, *P. aeruginosa*-infected, and rNDV-hBD4-MDBK injected, n = 6). Normal mice treated with PBS represented the control group (n = 2, data not shown). During 21 days of observation, mice in the B group experienced severe skin injuries (Figure 3B and C) and exhibited less physical activity, including reduced drinking and eating habits (data not shown). More severe skin damage was observed in the B/P mice (Figure 3B and C). Their burn wounds were larger than those of the B group and were colonized with the greatest number (2.44 × 10^3^ cfu/ml) of *P. aeruginosa* (Figure 3B). Mice treated with the rNDV-infected MDBK cells were also colonized with a similar number (2.43 × 10^3^ cfu/ml) of *P. aeruginosa* and experienced almost the same burn and bacterial infection sequelae as seen in those of the B/P group (Figure 3B and C). However, the B/P/H mice, which were treated with rNDV-hBD4-infected MDBK cells, showed the most rapid skin recovery (Figure 3B and C). Compared with the B, B/P, and B/P/N mice, *P. aeruginosa* was barely detected in the injured skin of the B/P/H mice (1.80 × 10^2^ cfu/ml; *p* < 0.001) (Figure 3B). At 21 days post-burn, only small skin contractures remained in the B/P/H mice as burn scars, and their skin had recovered as completely as the control mice (Figure 3B and C). These results suggest that hBD4 expressed from rNDV protects burn wounds from subsequent *P. aeruginosa* infection.

## Discussion

hBD4 is a known antimicrobial peptide [14-16,28]. However, it is difficult to utilize such a small peptide in currently available expression systems. We solved this problem by using the NDV vector. The NDV has been previously described as a highly useful vector for its oncolytic property in cancer therapy, stable expression efficiency as a vaccine platform, and relative safety in human applications because its RNA genome was not able to integrate into DNA chromosome [23,27]. These features make the NDV a better candidate than any other viral vectors in future clinical purposes.

Additionally, NDV itself has been known as a strong immune inducer [29]. However, treatment with NDV-infected MDBK cells did not benefit the burn-injured and subsequently *P. aeruginosa*-infected skin of mice in our model (Figure 3B and C). In our animal model, we complicated the burn wounds with *P. aeruginosa* infection, and only the mice treated with rNDV-hBD4-infected MDBK cells overcame the supplementary pseudomonal exacerbation (Figure 3B and C). The actual *P. aeruginosa* colony number was also extremely reduced only in the B/P/H mice (Figure 3B). With evident efficacy against *P. aeruginosa* (Figure 3A), these results imply that hBD4 can inhibit the growth of *P. aeruginosa* and intensify the rNDV-hBD4 effectiveness in burn wound therapy against the secondary bacterial complications.

MDBK cell-based application of hBD4 may be rejected for clinical application in humans. Due to this limitation, we also included human-origin cells including A549, HEp-2, and HDF cells in the initial test to select the best-fit cell candidate for the NDV-foreign gene expression (Figure 2A). However, all these human cells were insufficient for foreign gene expression, compared with MDBK cells, and excluded in further analysis. In this screening process, the rNDV-GFP virus which is very sensitive to the interferon (IFN) activation was used because the GFP expression can be dependent on the IFN level synthesized in the infected cells. Therefore, the GFP intensity might determine which cell line could be suitable for the foreign gene expression from the rNDV.

The direct application of hBD4 expression plasmid or rNDV-hBD4 virus into the burn-injured skin layers may be also considered. However, burn-injured skin may not effectively support hBD4 expression and may require aseptic surgical debridement for normal skin regeneration or replacement surgery [30]. Also, in severe cases of burn injury, the dermal skin layer can be affected, and destructions in nerves and blood vessels may result in subsequent bacterial colonization in the burn wounds [31]. Hence, we used an intermediate host, the MDBK cells, to express hBD4 in burn-injured skin layers. For the application of rNDV-hBD4 virus in human burn injuries, NDV-susceptible human cells or synthetic tissue scaffolds harboring them may need to be engineered for efficient hBD4 expression in burn wounds.

## Conclusions

Herein, we evaluated the therapeutic efficacy of the rNDV-hBD4 virus against *P. aeruginosa* infection in burn wounds. As an alternative clinical measure in burn wound remedies, hBD4 was applied to protect the burned skin of mice from *P. aeruginosa* complication, resulting in marked skin recovery. Although suitable cell lines or any equivalents, in addition to MDBK cells, should be further evaluated for the clinical application of rNDV-hBD4 in severe cases of burn injuries in humans, our results potentiate the therapeutic index of hBD4 and the use of the NDV vector in human cases of burn injury.

## Abbreviations

NDV: Newcastle disease virus
hBD4: Human β-defensin 4
GFP: Green fluorescent protein
cfu: Colony forming unit
